# Exogenous Application of Citric Acid Ameliorates the Adverse Effect of Heat Stress in Tall Fescue (*Lolium arundinaceum*)

**DOI:** 10.3389/fpls.2016.00179

**Published:** 2016-02-18

**Authors:** Longxing Hu, Zhifei Zhang, Zuoxiang Xiang, Zhijian Yang

**Affiliations:** Department of Turfgrass Sciences, College of Agronomy, Hunan Agricultural UniversityChangsha, China

**Keywords:** heat stress, tall fescue, citric acid, antioxidant enzymes, heat shock proteins (HSP)

## Abstract

Citric acid may be involved in plant response to high temperature. The objective of this study was to investigate whether exogenous citric acid could improve heat tolerance in a cool-season turfgrass species, tall fescue (*Lolium arundinaceum*), and to determine the physiological mechanisms of citric acid effects on heat stress tolerance. The grasses were subjected to four citric acid levels (0, 0.2, 2, and 20 mM) and two temperature levels (25/20 and 35/30 ± 0.5°C, day/night) treatments in growth chambers. Heat stress increased an electrolyte leakage (EL) and malonaldehyde (MDA) content, while reduced plant growth, chlorophyll (Chl) content, photochemical efficiency (*F*v/*F*m), root activity and antioxidant enzyme activities (superoxide dismutase, SOD; catalase, CAT; peroxidase, POD). External citric acid alleviated the detrimental effects of heat stress on tall fescue, which was evidenced by decreased EL and MDA content, and improved plant growth under stress conditions. Additionally, the reduction in Chl content, *F*v/*F*m, SOD, POD, CAT and root activity were ameliorated in citric acid treated plants under heat stressed conditions. High temperature induced the expression of heat shock protein (*HSP*) genes, which exhibited greater expression levels after citric acid treatment under heat stress. These results suggest that exogenous citric acid application may alleviate growth and physiological damage caused by high temperature. In addition, the exogenously applied citric acid might be responsible for maintaining membrane stability, root activity, and activation of antioxidant response and *HSP* genes which could contribute to the protective roles of citric acid in tall fescue responses to heat stress.

## Introduction

High temperature is becoming one of the major factors limiting the growth and development of cool-season turfgrass (Larkindale and Huang, 2004). Generally the optimal temperatures for the growth and development of cool-season turfgrass species range from 15 to 24°C for shoots and 10 to 18°C for the roots (Paulsen, 1994); however, in transitional and warm climatic regions, the temperature may peak to 38°C or higher during the summer. Therefore, the supraoptimal temperatures for prolonged periods often limit shoot and root growth, decline turf visual quality and root viability (Huang and Gao, 2000). In addition, high temperature would damage various physiological and metabolic processes that lead to premature leaf senescence (Liu and Huang, 2000; Huang et al., 2012). Heat stress induced senescence is associated with metabolic alterations as well as production of oxidative stress causing ROS in (Wahid et al., 2007). Oxidative stress can cause lipid peroxidation, membrane injury, protein degradation and enzyme inactivation in plants (Sairam et al., 2000; Meriga et al., 2004). However, plants have developed the enzymatic and non-enzymatic scavenging systems to scavenge the intra-cellar ROS (Liu and Huang, 2000). The enzymatic scavenging system includes SOD, which convert the superoxide radicals to hydrogen POD, and the CAT and PODs (POD), which trigger the conversion of H_2_O_2_ to water and oxygen (Mittler, 2002).

Heat stress remarkably affects protein metabolism (Monjardino et al., 2005; He and Huang, 2007). Under high thermal conditions, normal proteins and mRNAs synthesis are inhibited. Conversely, transcription and translation of a HSPs may be triggered or enhanced when plants are subjected to elevated temperature (Vierling, 1991). The HSPs are molecular chaperones, which enhances plant tolerance to extreme heat shock conditions. The chaperons act by protecting the native proteins from denaturation, and thus improving protein stability under stresses (Schoffl et al., 1999). Based on their approximate molecular weight, the principal *HSPs* are grouped into three major families: LMW-HSP that range from 15 to 30 kDa, HSP70 ranging from 69 to 71 kDa and HMW-HSP ranging from 80 to 114 kDa (Mian et al., 2008). There is sufficient evidence that HSPs play crucial roles in thermotolerance, and that some specific HSPs causally participate in the acquisition of thermotolerance capacity (Wang et al., 2014). *LMW-HSP* genes such as *ApHsp26.2* and *ApHsp26.7a* have been confirmed to accumulate highly in heat-tolerant creeping bentgrass (Wang and Luthe, 2003). In addition, the superior thermo-tolerance in higher plants was correlated with *LMW-HSP* (*HSP18.1*, *HSP17.9*) expression in wheat (Basha et al., 1999) and with *HMW-HSP* (*HSP101*) expression in *Arabidopsis* (Queitsch et al., 2000).

Citric acid is one of the TCA intermediates, which serves as the source of carbon skeleton and cellular energy, which are utilized in the respiratory cycle and other biochemical pathways (da Silva, 2003). Citric acid as a vital organic acid, has been reported to be closely related with aluminum poisoning (Ma and Furukawa, 2003), iron stress (Shlizerman et al., 2007), heavy metal stress tolerance (Gao et al., 2010) and salinity stress (Sun and Hong, 2011). In addition, citrate complex is one of the mobile forms of iron that participate in iron transportation inside plants (Hell and Stephan, 2003). Additionally, the complex has been confirmed to increase vase life and Chl content of tuberose and *Lilium* (Darandeh and Hadavi, 2011). Previously, we reported that heat stress altered the leaf metabolic profiles of a cool-season grass species, tall fescue (*Festuca arundinacea*), and demonstrated that the citric acid served majorly as an antioxidant respiratory metabolism intermediate involving the defense pathways in response to high temperature (Zhao et al., 2015).

Tall fescue (*F. arundinacea* Schreb) is one of the widely used cool season forage and turfgrass species because of its adaptability, yield and persistence. Natural populations are scattered from Northern Europe including the Mediterranean region to North Africa, the Middle East, central Asia and Siberia. The optimum growth temperature of tall fescue ranges from 15 to 25°C. High temperatures of over 35°C in warm climate regions during summer are usually detrimental to turf quality and growth. Many approaches have been applied to improve whole-plant stress tolerance in cool-season turfgrass species, including exogenous application of cytokinins and ascorbic acid (Xu and Huang, 2009; Kumar et al., 2011), Ca^2+^ (Jiang and Huang, 2001a) and salicylic acid (He et al., 2005). Previously, endogenous citric acid have been reported in response to heat stress, and mainly served as antioxidant and intermediate in respiration metabolisms involving the defense pathways in adaptation to high temperature (Zhao et al., 2015). However, there is unreliable evidence regarding the protective roles of exogenous citric acid on cool season turfgrass species under heat stress conditions.

The objective of this study was to investigate if the exogenously applied citric acid improve tolerance to heat stress by modulation of plant growth, leaf senescence, root activity, antioxidant capacity and HSP gene expressions in the cool-season grass tall fescue.

## Materials and Methods

### Plant Materials and Growth Conditions

Twenty-five seeds of tall fescue ‘Bar Lexus’ were sowed in plastic pots (9-cm upper diameter, 7-cm lower diameter, 12-cm height) filled with peat soil and sand with the ratio of 1:1 (v/v). After germination, 15 uniform seedlings were allowed to grow and watered every other day with tap water. All plants were placed in a controlled walk-in growth room for 50 days with the temperature of 25/20 ± 0.5°C (day/night), a 12-h photoperiod, and a PAR of 240 μmol m^-2^ s^-1^ at the canopy level. Turf was hand-clipped at 6-cm canopy height three times per week. Plants were fertilized weekly with 300 ml of half-strength Hoagland nutrient solution per pot (Hoagland and Arnon, 1950). After 50 days of growth, plants were transferred to growth chambers (HP1500 GS-B; Wuhan Ruihua Instrument & Equipment, Wuhan, China) for 5 days before treatments were imposed with the temperature of 25/20 ± 0.5°C (day/night), a 12-h photoperiod, and a PAR of 400 μmol m^-2^ s^-1^ at the canopy level.

### Treatment and Experiment Design

Plants were subjected to the following two temperature treatments for 15 days: control: plants were maintained at the optimal temperature (25/20 ± 0.5°C, day/night); heat stress: plants were exposed to supraoptimal temperatures of 10°C above the optimum range (35/30 ± 0.5°C, day/night). All plants were watered daily to maintain the soil water content at field capacity. The two temperature treatments were repeated three times on three different sets of plants. All growth chambers had a 12-h photoperiod, PAR of 400 μmol m^-2^ s^-1^, and 70 to 80% relative humidity.

Citric acid treatments were applied to the plants 3 days before the imposition of high temperature treatment, and then applied weekly during the period of temperature treatments. For citric acid treatment, plants in each plastic pot were foliar sprayed with 20 ml of 0, 0.2, 2, and 20 mM sodium citrate solution (Sigma–Aldrich, St. Louis, MO, USA) until the turf grass canopy was saturated and some dripping occurred. Treatments were arranged as a split-plot design with temperature treatments as main plots and citric acid treatments as subplots. Each temperature and citric acid treatment had four replicates. All pots were randomized within the growth chamber.

After 15 days of heat stress, the shoots and roots were harvested for physiological analysis.

### Measurements

Vertical shoot growth rate was estimated by measuring the difference in average turf canopy height before and after treatment using a ruler according to the method described by Hu et al. (2012). After 15 days of treatment, both shoots and roots were harvested separately and dry weights were recorded.

To determine EL, 0.1 g fully expanded leaves were washed three times with deionized water and placed in tubes filled with 15 ml deionized water after cut to 0.5 cm long segments, and then shaken for 24 h. The initial conductivity (Ci) was determined using a conductivity meter (JENCO-3173, Jenco Instruments, Inc., San Diego, CA, USA). The tubes were autoclaved at 121°C for 20 min, and the conductivity of the incubation solution with killed tissues (*C*_max_) was determined after the solution cooled to room temperature. Relative EL was calculated using the formula: EL (%) = (*C*i/*C*_max_) × 100.

Leaf Chl content was measured by using a hand-held Chl meter (SPAD-502 Plus, Minolta Corp., Spectrum Technologies, Inc.). The measurement points were randomly selected on the third fully developed leaves from top, and four points were selected on each leaf for all leaves in one pot (Richardson et al., 2002).

Leaf *F*v/*F*m (maximum quantum efficiency of PSII) was estimated by measuring the ratio of variable to maximum fluorescence of Chl (*F*v/*F*m) with a Chl fluorometer (Walz, Effeltrich, Germany). Measurements were made on intact leaves with the fluorometer after plants were adapted in darkness for 30 min.

Root activity was determined by the TTC method (Clemensson-Lindell, 1994). Briefly, 0.5 g fresh root was immersed in 10 ml of equally mixed solution of 0.4% TTC and phosphate buffer, and kept in the dark at 37°C for 2 h. Subsequently, 2 ml of 1 M H_2_SO_4_ was added to stop the reaction with the root. The root was dried with filter paper and then extracted with ethyl acetate. The red extract was transferred into the volumetric flask to reach 10 ml by adding ethyl acetate. The absorbance of the extract at 485 nm was recorded. Root activity was expressed as TTC reduction intensity. Root activity = amount of TTC reduction (μg)/fresh root weight (g) × time (h).

For the determination of SOD, POD, CAT activity and MDA content, about 0.3 g of fully developed leaves was sampled at 15 days of treatment, frozen immediately with liquid nitrogen, and then stored at -80°C for subsequent analysis. Frozen leaves were homogenized in 50 mM ice-cold phosphate buffer (pH 7.0) with a pre-chilled mortar and pestle. The homogenate was centrifuged at 12,000 × *g* for 15 min at 4°C. The supernatants were collected to determine soluble protein, enzyme activities, and MDA content. Protein content was quantified using the method described by Bradford (1976).

The SOD activity was determined using the method described by Hu et al. (2012). Briefly, the color reaction contained 50 mM of sodium phosphate buffer (pH 7.0), 1.125 mM NBT, 1.3 mM of riboflavin (7,8- dimethyl-10-ribitylisoalloxazine), 13 mM of methionine, 75 nM of EDTA and 100 ml of enzyme extract with non-enzyme solution as control. The reaction mixtures were illuminated under a set of 40-W fluorescent tubes (Philips, Amsterdam, Netherlands) for 20 min. The changes of absorbance at 560 nm were determined with a spectrophotometer (UV-2600). One unit of enzyme activity was defined as the amount of enzyme required to inhibit the NBT reduction by 50%.

The activities of CAT and POD were measured using the method of Chance and Maehly (1955). For CAT, the decomposition of H_2_O_2_ was measured by the decline in absorbance at 240 nm for 1 min. The 3-ml reaction mixture contained 50 mM phosphate buffer (pH 7.0), 15 mM H_2_O_2_, and 0.1 ml enzyme extract, which initiated the reaction. For POD, the oxidation of guaiacol was measured by the increase in absorbance at 470 nm for 1 min. The reaction mixture contained 50 μl of 20 mM guaiacol, 2.8 ml of 10 mM phosphate buffer (pH 7.0), and 0.1 ml enzyme extract. The reaction was started with 20 μl of 40 mM H_2_O_2_.

The MDA content was determined by the TBA reaction using the method described by Heath and Packer (1968). One milliliter of extract was mixed with 1 ml of reaction solution containing 20% (v/v) trichloroacetic acid and 0.5% (v/v) TBA. The mixture was heated in a 95°C water bath for 30 min, then cooled to room temperature, and centrifuged at 10,000 *g* for 10 min. The supernatant was read for absorbance at 532 and 600 nm. The absorbance for non-specific absorption at 600 nm was subtracted from the value at 532 nm. The content of MDA was calculated using the adjusted absorbance and the extinction coefficient of 155 mM^-1^cm^-1^ (Heath and Packer, 1968).

Leaf citric acid content was determined according to the high-performance liquid chromatography (HPLC) method described by Shui and Leong (2002) with slight modifications. Briefly, 0.5 g of fresh samples was ground into fine powder in liquid nitrogen, and then homogenized in 5 ml ultra-pure water. The homogenate was centrifuged at 5000 rpm for 5 min at room temperature. The supernatants were filtered through a 0.22 μm membrane filter and inject 10 μl of volume to the reversed-phase HPLC (RP-HPLC) with an Agilent 1100 Series (Agilent Technologies, Atlanta, GA, USA) according to the Hu et al. (2015a). The citric acid was identified based on retention time and quantified based on the UV spectra relative to the standards.

Total RNA was extracted from fresh tissues by using Trizol reagent (Invitrogen, Carlsbad, CA, USA) according to the user manuals. After extraction, the RNA pellet was dissolved in 100 μl of RNase-free water. RNase-free DNase I was added to the total RNA to remove DNA contamination. The total RNA concentration was then determined by absorbance at 260 nm and RNA quality was evaluated on a 0.8% agarose gel. The first strand cDNA fragments were synthesized from 2 μg of total RNA using oligo(dT)12-18 primer using cDNA synthesis kit (Fermentas, Burlington, Ontario, Canada) according to the user manual. Gene-specific primers (**Table 1**) were designed based on the target gene sequences using Primer 5 software (Hu et al., 2015b). *YT521-B* gene was used as internal standard. The qRT-PCRs were performed with ABI7500 in a final volume of 20 μl, with each containing 2 μl of cDNA, 10 μl of 2x SYBR Green qPCR Mix (Takara, Otsu, Shiga, Japan) and 2 μM of the forward and reverse primers. Three independent biological replicates of each sample and two technical replicates of each biological replicate were used for real-time PCR analysis. The thermal cycling conditions were as follows: 40 cycles of 95°C denaturation for 5 s, and 52–55°C annealing and extension for 20 s. After the PCR, a melting curve was generated by gradually increasing the temperature to 95°C to test the amplicon specificity. To determine relative fold differences for each sample, the CT value for each gene was normalized to the CT value for the reference gene and was calculated relative to a calibrator using the DDCT method as described by Livak and Schmittgen (2001).

**Table 1:** Primer sequence used for real-time quantitative PCR.

Gene name	Accession	Primers sequences (5′-3′)	Size (bp)	Tm (°C)
*HMW-HSP*	DT697638	F TTGCAATAGATCGCAGTCAGGAG	113	55
		R CACGAGGTGAGAGTTCGCATAAT		
*HSC70*	CK800824	F CATCATCGGTTTCGACAACATCC	187	55
		R CCACGTCCAACATAAGCACAAGA		
*OsHSP74.8*	AK073817	F CGAGCAGTTCGAGTACCAGG	465	58
		R TCAGCCATAGCTTCCCATAC		
*LMW-HSP*	DT696482	F TCTGATCGTCTGCCATTTCCTA	109	55
		R GCCCGTGGTCAATCTCCTC		
*YT521-B*		F TGTAGCTTGATCGCATACCC	122	55
		R ACTCCCTGGTAG CCACCTT		


### Statistical Analysis

All data were subjected to two-way ANOVA (analysis of variance) according to the general linear model procedure of SAS (SAS Institute, Cary, NC, USA) to determine the effects of temperature, citric acid and their interactions. Treatment means were separated using the Duncan’s multiple range test at the *P* = 0.05 level of probability.

## Results

### High Temperature and Exogenous Citric Acid Induced Accumulation of Endogenous Citric Acid in Leaves of Tall Fescue

**FIGURE 1:**
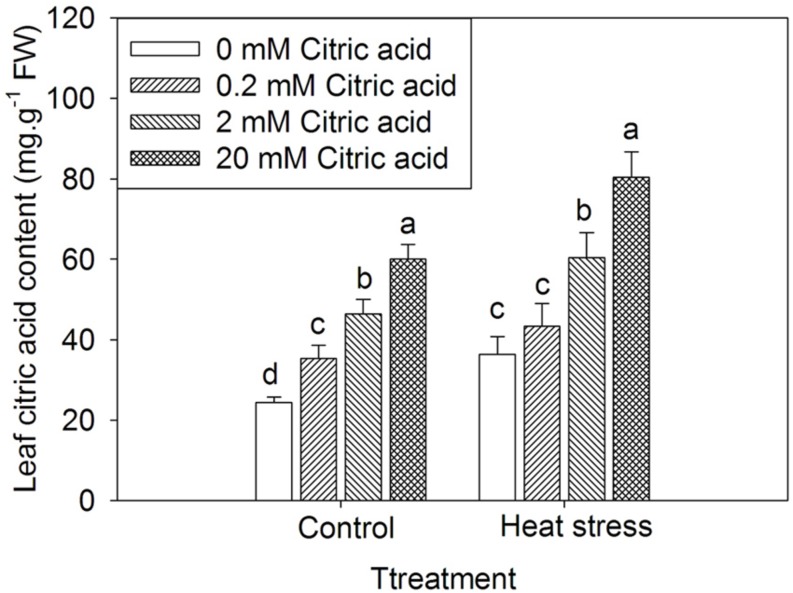
**Effect of exogenous citric acid on endogenous citric acid content under control (25/20 ± 0.5°C, day/night) and heat stressed (35/30 ± 0.5°C, day/night) conditions in tall fescue.** Vertical bars on the top provide the standard deviation (*n* = 4), and bars with the same letter indicate no significant difference at *P* < 0.05 for the comparison of different citric acid concentrations under control or heat stress.

In this study, to investigate the relationship between heat stress and citric acid content, the endogenous citric acid levels in leaves of tall fescue were quantified after treatments with high temperature (35/30 ± 0.5°C, day/night) for 15 days. In tall fescue leaves, heat stress significantly increased endogenous citric acid content when compared to the control plants (**Figure 1**). This result indicated that citric acid might be involved in heat stress response. Additionally, treatments with higher concentrations of citric acid resulted in higher accumulation of endogenous citric acid in both control and heat stressed plants, and the accumulation of citric acid content by exogenous application of citric acid was in a concentration-dependent manner (**Figure 1**).

### Exogenous Citric Acid Improved Heat Stress Tolerance in Tall Fescue

**FIGURE 2:**
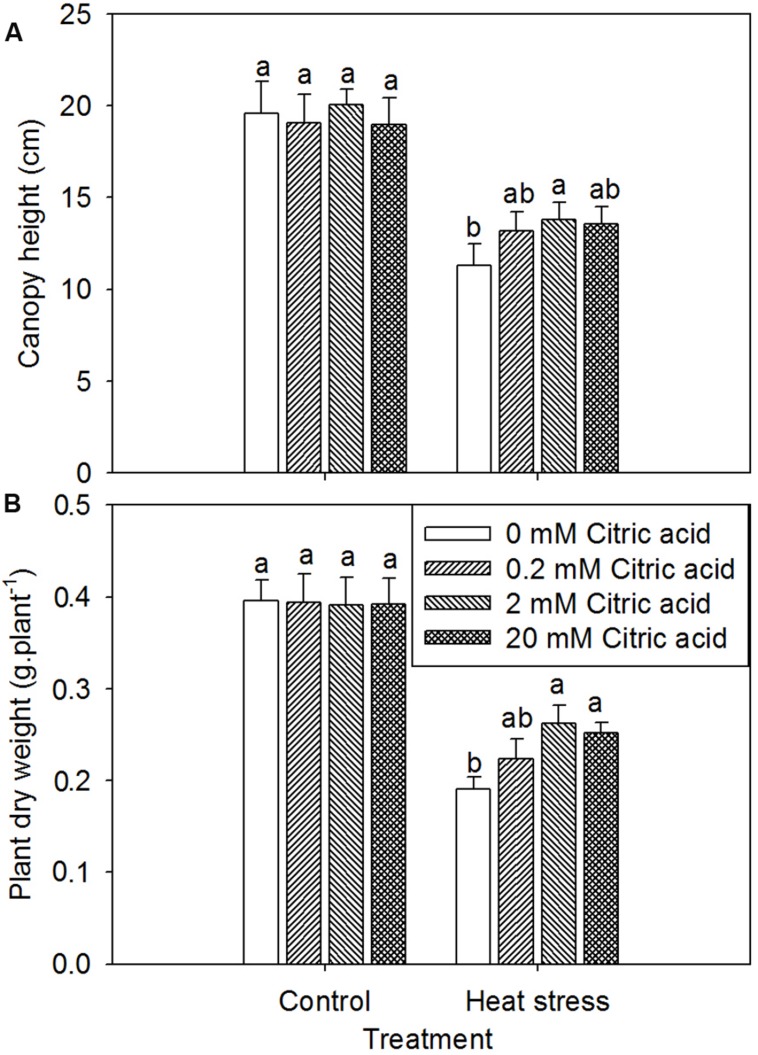
**Effect of exogenous citric acid on canopy height **(A)** and plant dry weight **(B)** under control (25/20 ± 0.5°C, day/night) and heat stressed (35/30 ± 0.5°C, day/night) conditions in tall fescue.** Vertical bars on the top provide the standard deviation (*n* = 4), and bars with the same letter indicate no significant difference at *P* < 0.05 for the comparison of different citric acid concentrations under control or heat stress.

Under optimum growth conditions, tall fescue growth was generally equivalent to the non-citric acid treated plants irrespective of foliar concentration of citric acids, as showed by the canopy height and plant dry weight (**Figure 2**). High temperature remarkably reduced plant growth in tall fescue as indicated by the lower canopy height and plant dry weight (**Figure 2**). However, the citric acid treatment alleviated the decline in canopy height and plant dry weight under heat stress as compared to the non-treated plants, particularly at 2 mM concentration.

### Exogenous Citric Acid Alleviated the Damage Effect of Heat stress on Tall Fescue

**FIGURE 3:**
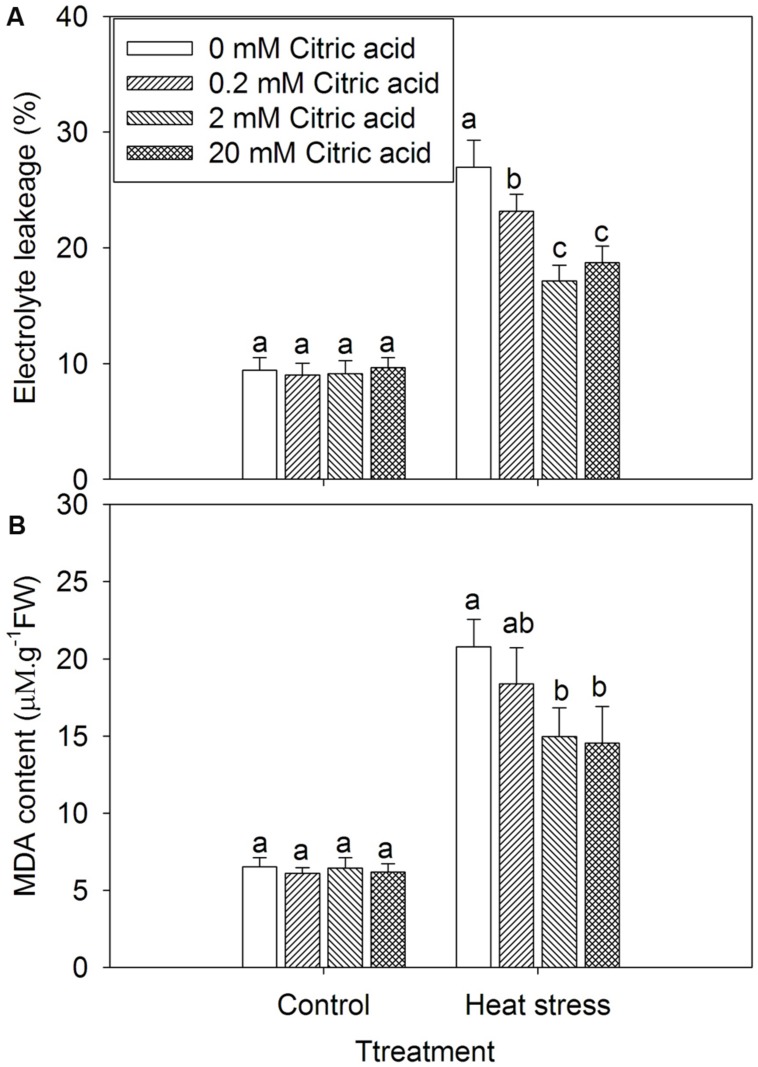
**Effect of exogenous citric acid on EL **(A)** and MDA content **(B)** under control (25/20 ± 0.5°C, day/night) and heat stressed (35/30 ± 0.5°C, day/night) conditions in tall fescue.** Vertical bars on the top provide the standard deviation (*n* = 4), and bars with the same letter indicate no significant difference at *P* < 0.05 for the comparison of different citric acid concentrations under control or heat stress.

Leaf EL and MDA levels were generally employed to assess the extent of membrane damage caused by environmental stress. Treatment with citric acid alone had no effect on the leaf EL and MDA content under control growth conditions (**Figure 3**). Heat stress treatment significantly increased EL and induced the accumulation of MDA in leaves of tall fescue (**Figure 3**), while citric acid treated plants displayed significantly lower EL and MDA content in comparison with non-treated plants (**Figure 3**).

### Effect of Exogenous Citric Acid on Leaf Senescence and Photochemical Efficiency in Tall Fescue Under Heat Stress

**FIGURE 4:**
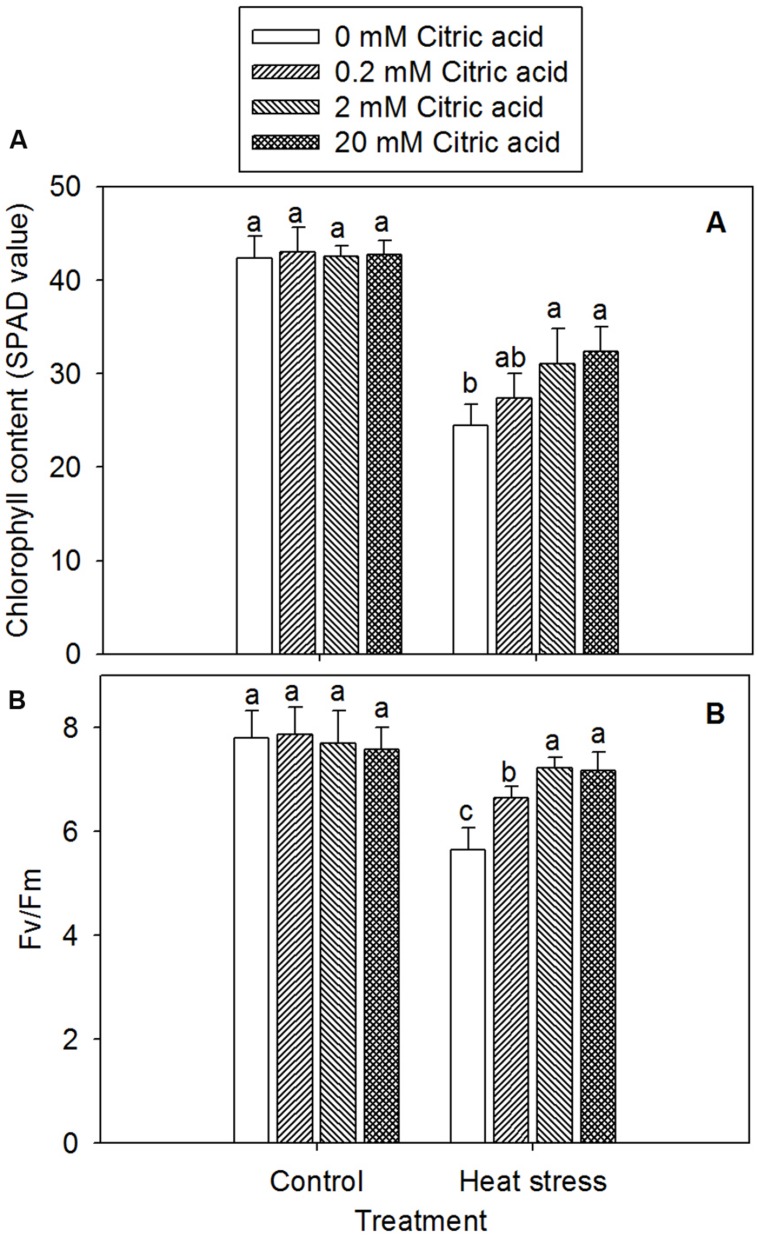
**Effect of exogenous citric acid on chlorophyll content (SPAD values; **A)** and (*F*v/*F*m; **B)** under control (25/20 ± 0.5°C, day/night) and heat stressed (35/30 ± 0.5°C, day/night) conditions in tall fescue.** Vertical bars on the top provide the standard deviation (*n* = 4), and bars with the same letter indicate no significant difference at *P* < 0.05 for the comparison of different citric acid concentrations under control or heat stress.

Leaf senescence was assessed from a decline in the Chl content based on a SPAD values. The *F*v/*F*m of photosynthesis II (PSII, *F*v/*F*m) was measured by analysis of Chl fluorescence. A significant decline in Chl content and *F*v/*F*m in leaves of tall fescue was observed under heat stress, while treatment with different concentration of citric acid alone had no effect under control condition (**Figures 4A,B**). Under stress condition, however, citric acid treated plants displayed an increased Chl content and *F*v/*F*m, particularly for the concentration of 2 and 20 mM, when compared to the 0 mM citric acid in tall fescue (**Figures 4A,B**).

### Effect of Exogenous Citric Acid on Root Activity in Tall Fescue Under Heat Stress

**FIGURE 5:**
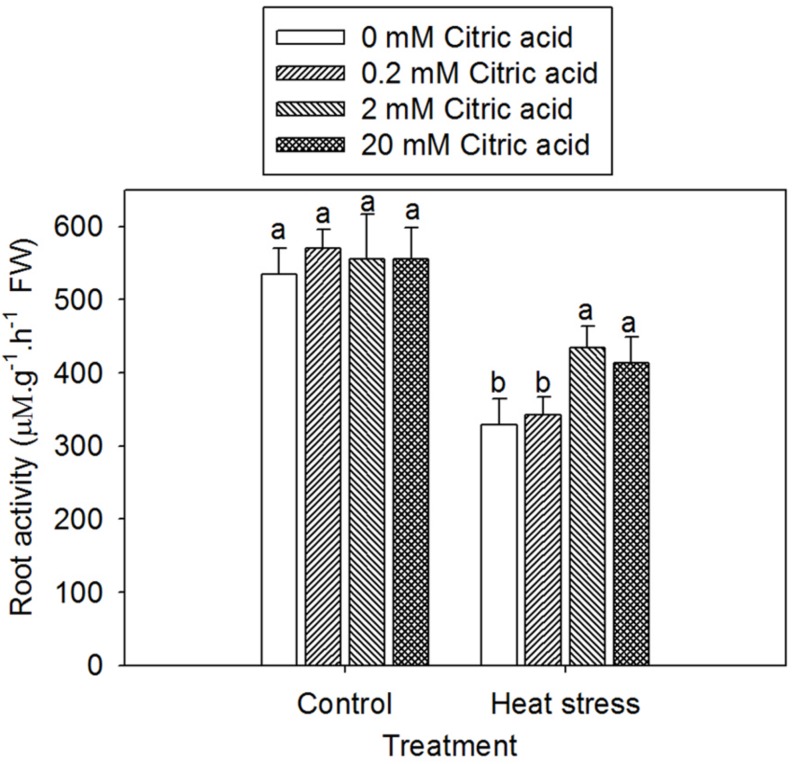
**Effect of exogenous citric acid on root activity under control (25/20 ± 0.5°C, day/night) and heat stressed (35/30 ± 0.5°C, day/night) conditions in tall fescue.** Vertical bars on the top provide the standard deviation (*n* = 4), and bars with the same letter indicate no significant difference at *P* < 0.05 for the comparison of different citric acid concentrations under control or heat stress.

Reduction of TTC has been used frequently employed to assess the root metabolic activities in under various growth conditions. Heat treatment with or without citric acid addition resulted in a decreased root activity when compared to the control plants, irrespective of citric acid concentrations (**Figure 5**). Heat treatment plus citric acid at 2 and 20 mM increased root activity by 32 and 25%, respectively, when compared with heat treat treatment alone. No significant difference among the four citric acid application levels was observed under control conditions (**Figure 5**).

### Effect of Exogenous Citric Acid on Antioxidant Activities in Tall Fescue Under Heat Stress

**FIGURE 6:**
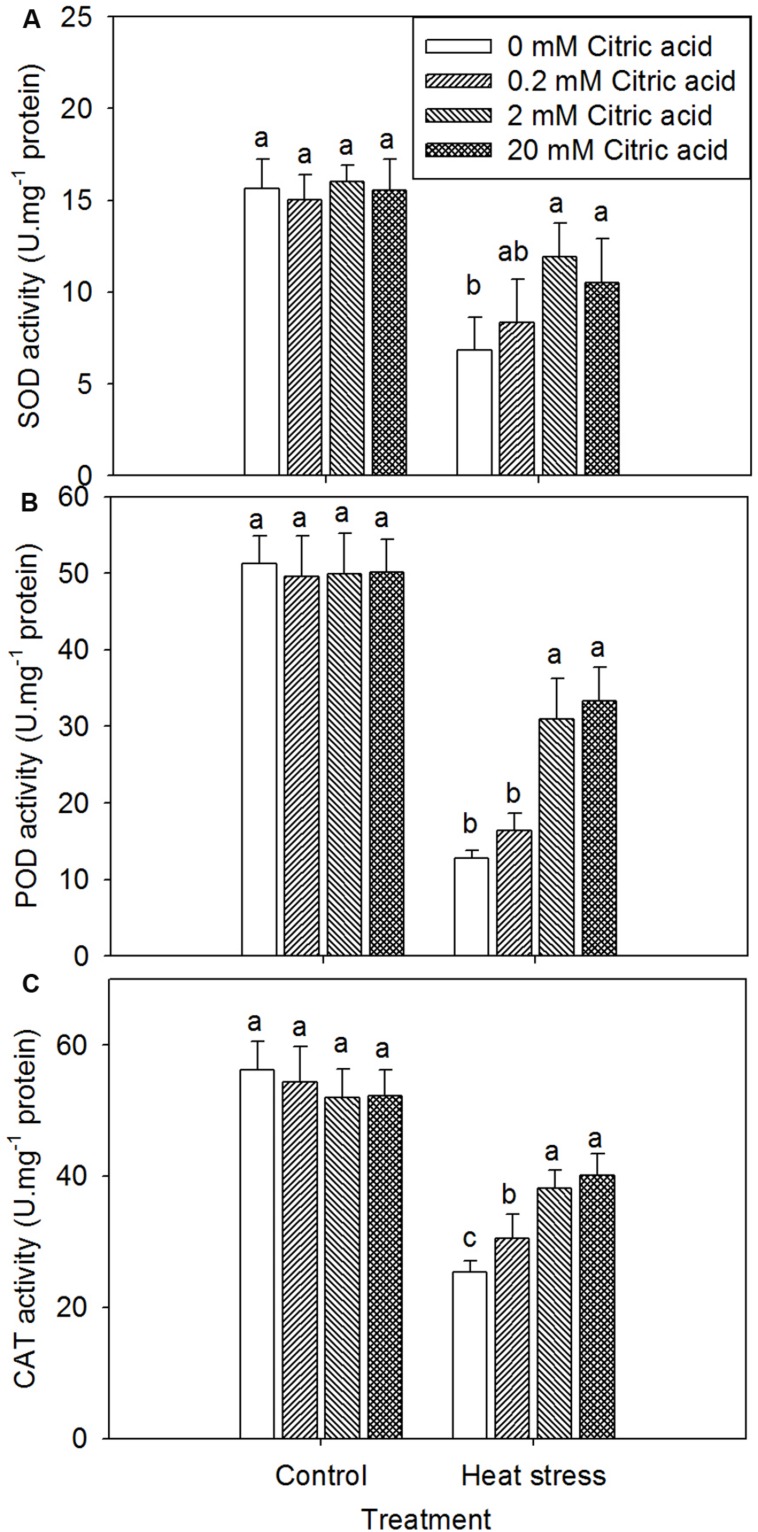
**Effect of exogenous citric acid on SOD **(A)**, POD **(B)** and CAT **(C)** under control (25/20 ± 0.5°C, day/night) and heat stressed (35/30 ± 0.5°C, day/night) conditions in tall fescue.** Vertical bars on the top provide the standard deviation (*n* = 4), and bars with the same letter indicate no significant difference at *P* < 0.05 for the comparison of different citric acid concentrations under control or heat stress.

On one hand, foliar application of exogenous citric acid alone had no effect on SOD, POD, and CAT activity under control condition (**Figures 6A–C**). Heat treatment led to the deactivation of these antioxidant enzymes irrespective of citric acid concentration, when compared to the control plants (**Figures 6A–C**). On the other hand, foliar application of exogenous citric acid improved the activities of SOD, POD, and CAT under heat stress, when compared to the non-citric acid treated plants, particularly with the concentration of 2 and 20 mM (**Figures 6A–C**).

### Effect of Exogenous Citric Acid on the Expression of Heat Shock Proteins in Tall Fescue Under Heat Stress

**FIGURE 7:**
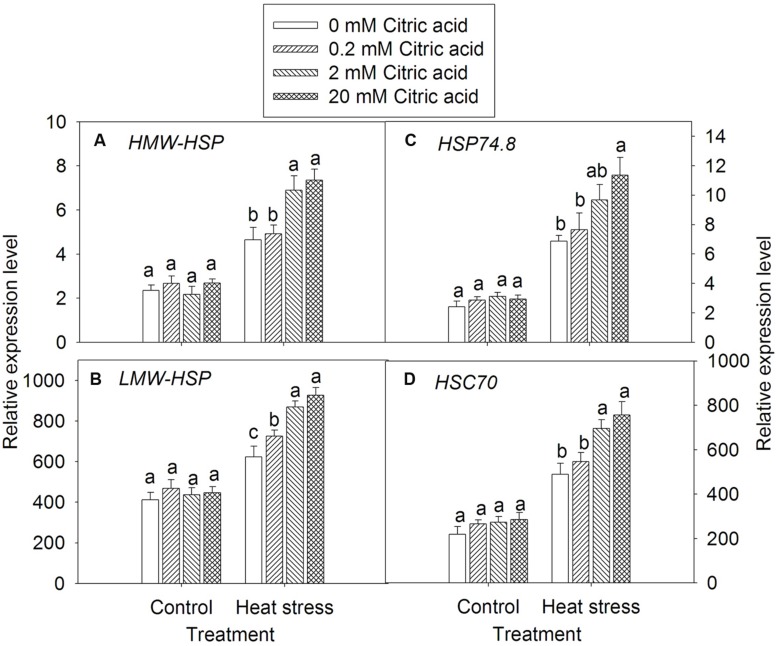
**Effect of exogenous citric acid on the expression of heat shock protein (*HSP*) gene family under control (25/20 ± 0.5°C, day/night) and heat stressed (35/30 ± 0.5°C, day/night) conditions in tall fescue.** **(A)** *HMW-HSP;* **(B)** *LMW-HSP;* **(C)** *HSP74.8;* **(D)** *HSC70*; Vertical bars on the top provide the standard deviation (*n* = 4), and bars with the same letter indicate no significant difference at *P* < 0.05 for the comparison of different citric acid concentrations under control or heat stress.

Exogenously applied citric acid alone had no significant effect on the expression levels of HSP gene families, including HMW-HSP, LMW-HSP, HSP74.8, and HSC70 under control condition (**Figures 7A–D**). Heat stress induced higher expression levels of HSP HMW-HSP, LMW-HSP, HSP74.8, and HSC70 genes when compared to the control plants. Evidently, foliar application of 2 and 20 mM concentration of exogenous citric acid significantly induced HSP gene expressions, when compared to the 0 and 0.2 mM concentration of citric acid under heat stress condition (**Figures 7A–D**).

## Discussion

Our previous study demonstrated that heat stress substantially increased endogenous citric acid levels in tall fescue, and may be involved in high temperature adaptation (Zhao et al., 2015). Concurrently, other studies suggested that exogenous citric acid could promote growth and improve Cd and salinity stress tolerance in plants (Gao et al., 2010; Sun and Hong, 2011). In this study, the positive role of citric acid on tall fescue response to heat stress was assigned via exogenously applied citric acid. The results of this study indicated that exogenous application of citric acid alleviated detrimental effects of heat stress on tall fescue; this may be a practical approach to improve heat stress tolerance in cool-season turfgrass species during summer seasons.

Heat stress often triggers drastic changes in the cell membrane stability, and ultimately influences the sensors present in the membrane (Kumar et al., 2013). It is generally accepted that the maintenance of cell membrane integrity and stability under stress conditions is critical for plant survival (Blum and Ebercon, 1981). Marcum (1998) has shown that cell membrane stability was positively correlated to turf quality and can be used to predict the whole-plant heat tolerance in Kentucky bluegrass cultivars. The level of lipid peroxidation, expressed as MDA content, has been used as an indicator of free radical damage to cell membranes (Smirnoff, 1995). No significant changes in EL and MDA content were observed after citric acid treatment for tall fescue under control conditions (**Figure 3**). However, leaf EL and MDA in tall fescue increased under heat stress, but exogenous application of citric acid reduced lipid peroxidation, as indicated by the less accumulation of MDA and lower EL when compared to the untreated plants (**Figure 3**).These results suggested that application of citric acid would reduce loss of grasses under high temperature environments by mitigating oxidative stress.

In many plant species heat stress induces oxidative stress and alters the antioxidant activities of SOD, POD, and CAT enzymes (Jagtap and Bhargava, 1995; Gong et al., 1997), including turfgrass (Liu and Huang, 2000; Jiang and Huang, 2001a,b). SOD is responsible for catalyzing O_2_^-^ into H_2_O_2_ in plants (Miller et al., 2010; Sharma et al., 2013). The increased SOD activity was considered a protective mechanism against the formation of superoxide under stress conditions (Ostrovskaya et al., 2009). In this study, the SOD activity decreased under high temperature stress in tall fescue, this observation was consistent with the previous studies that revealed a decrease in SOD activities in cool-season turfgrass (Du et al., 2009; He and Huang, 2010). The alleviation of the SOD activity reduction by exogenously applied citric acid in tall fescue under heat stress may have resulted in less production of O_2_^-^ and thus lower heat induced oxidative damages in leaves.

Peroxidase and CAT are the major enzymes that convert H_2_O_2_ into H_2_O, which are produced through the dismutation of O_2_^-^ catalyzed by SOD (Miller et al., 2010; Sharma et al., 2013). The maintenance of higher POD and CAT activity may provide further oxidative protection by detoxifying H_2_O_2_ induced by heat stress through weakening the SOD enzyme system. In the present study, a decrease in POD and CAT activity were observed under heat stress conditions. These result suggested that high temperature triggered oxidative damage in leaves of tall fescue. The result further indicated that POD and CAT could be sensitive to high temperatures (Jiang and Huang, 2001a; He and Huang, 2010). Decrease in POD activity during heat stress have also been observed in other species, such as wheat (Almeselmani et al., 2006), rice (Shi et al., 2006), and mustard (Dat et al., 1998). In current study, external citric acid treatment helped to maintain higher POD and CAT activities when compared to the untreated plants under heat stress. These results indicated that the enhanced POD and CAT activities by exogenously applied citric acid might contribute to the reduced accumulation of MDA, H_2_O_2_, and O_2_^-^ contents, and alleviate the damage to cell membranes.

Roots play a critical role in plant tolerance to elevated temperatures. It has been reported that the significance of these vital organs in plant adaptation to heat stress is associated with their participation in water and nutrient uptake, as well hormone synthesis affecting shoot growth and development (Jiang and Huang, 2001a). The detrimental effects of heat stress on the plant roots are typically characterized by a changes in root morphology as well as root metabolic activities (Xu and Huang, 2000; Huang and Liu, 2003). In the present study, high temperatures significantly declined root activity along with a great reduction in shoot growth, suggesting that heat stress caused injurious effect on root growth and eventually preceded shoot growth inhibition in tall fescue, as indicated in previous studies (Du et al., 2009; Zhao et al., 2015). We also observed that exogenous application of citric acid alleviated the decrease in root activity and shoot growth (**Figures 2** and **5**). These results suggested that the exogenous citric acid ameliorated the heat-induced injury to plants. This could be possibly attributed to the improvement of root viability and functions, which were associated with water and nutrient uptake and transport to shoots (Huang and Xu, 2000) as well as the synthesis of phytohormones in roots (Liu et al., 2002).

High temperatures during summer often cause leaf senescence and eventually decline in turf quality in cool-season turfgrass (Jiang and Huang, 2001b). Leaf Chl content is a good indicator of leaf senescence and an important factor determining photosynthetic capacity. Many have demonstrated that high temperatures result in declines in Chl content in cool-season turfgrasses, contributing to leaf senescence and photosynthesis inhibition under heat stress (Liu and Huang, 2000; Jiang and Huang, 2001b; Zhang et al., 2010). In this study, heat stress caused a decrease in Chl content and the maximum quantum yield of photosynthesis system II (*F*v/*F*m). However, exogenous application of citric acid resulted in the alleviation of Chl and *F*v/*F*m reduction in leaves of tall fescue under heat stress. These results suggested that external citric acid treatment might have been related to the inhibition and loss of Chl under heat stress, which damaged the reaction centers (Kyle, 1987). These changes improved the photosynthetic capacity by reducing photo-oxidation (Wise and Naylor, 1987) or maintaining photosynthetic membrane integrity (Coria et al., 1998).

The HSPs are molecular chaperones which played an important role in the heat shock tolerance under high temperatures conditions (Schoffl et al., 1999). The presence and role of HSPs in heat tolerance has been examined in various crops, such as rice (*Oryza* sp.), wheat (*Triticum* sp.), barley (*Hordeum* sp.) and oat (*Avena* sp.) (Xu et al., 2011). Some studies have reported that changes in specific HSP expressions are related to heat stress tolerance in cool-season turfgrass species, such as creeping bentgrass (*Agrostis stolonifera*) and fescues (*Festuca* sp.) (Xu et al., 2011). In this study, high temperature induced HSP family genes expressions in tall fescue, suggesting that up-regulation of HSPs, primarily LMW-HSP, HMW-HSP, HSC70, or HSP74.8 based on their molecular weights, is a typical response of tall fescue to heat stress. However, exogenous application of citric acid induced a higher expression of these HSP genes under high temperatures in tall fescue, especially under high citric acid concentrations. These results suggested that the enhanced expression of this chaperone gene under heat stress as a result of exogenous application of citric acid is vital for heat tolerance in cool-season tall fescue (Tian et al., 2009; Xu et al., 2011).

**FIGURE 8:**
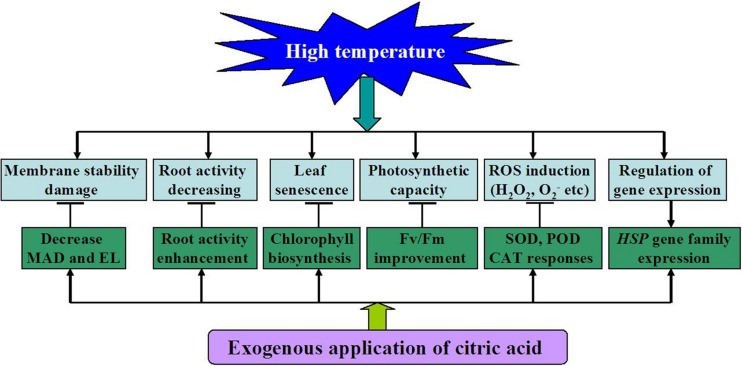
**A proposed model for citric acid-mediated high temperature stress responses in tall fescue**.

Based on the above observations, a model for citric acid-mediated heat stress response in tall fescue was proposed in this study (**Figure 8**). On one hand, heat stress had detrimental effects on the cell membrane, root activity, induced leaf senescence and ROS production. On the other hand, exogenous application of citric acid could promote antioxidant enzyme activities (SOD, POD, and CAT) and HSP family gene expressions, improve Chl biosynthesis and photosynthetic capacity. All these changes in turn alleviated cell membrane damages, ROS accumulation, leaf senescence and increased root activity all induced by high temperature.

## Conclusion

Heat stress and external application of citric acid induced the accumulation of endogenous citric acid levels in tall fescue leaf tissues. Heat stress was detrimental to plant growth, cell membranes, root activity, and antioxidant enzyme activities in tall fescue. However, external application of citric acid alleviated the detrimental effects of heat stress on tall fescue. The beneficial effect of external application of citric acid in tall fescue in response to heat stress may attribute to its protective effects on peroxidation-linked membrane deterioration, free radicals scavenging, maintenance of membrane stability, increase in root activity and activation of antioxidant response and HSP genes.

## Author Contributions

LH and ZY conceived and designed the experiment. LH and ZZ conducted the experiment. ZX and LH analyzed data, LH and ZY wrote the manuscript. The authors read and approved the paper.

## Conflict of Interest Statement

The authors declare that the research was conducted in the absence of any commercial or financial relationships that could be construed as a potential conflict of interest.

## Abbreviations

CAT: catalase
Chl: chlorophyll
EDTA: ethylene diaminetetra acetic acid
EL: electrolyte leakage
*F*v/*F*m: photochemical efficiency
*HSP*: heat shock protein
*HMW-HSP*: high molecular weight-HSP
*HSP74.8*: heat shock protein 74.8
*HSC70*: heat shock protein cognate 70
*LMW-HSP*: low molecular weight-HSP
MDA: malondialdehyde
NBT: nitro blue tetrazolium
PAR: photosynthetically active radiation
POD: peroxidase
ROS: reactive oxygen species
SOD: superoxide dismutase
TBA: thiobarbituric acid
TTC: triphenyl tetrazolium chloride
